# Chitosan Membranes Containing Plant Extracts: Preparation, Characterization and Antimicrobial Properties

**DOI:** 10.3390/ijms24108673

**Published:** 2023-05-12

**Authors:** Luiza Madalina Gradinaru, Mihaela Barbalata-Mandru, Alin Alexandru Enache, Cristina Mihaela Rimbu, Georgiana Ileana Badea, Magdalena Aflori

**Affiliations:** 1 “Petru Poni” Institute of Macromolecular Chemistry, 41A Grigore Ghica Voda Alley, 700487 Iasi, Romania; gradinaru.luiza@icmpp.ro (L.M.G.); mihaelamandru84@gmail.com (M.B.-M.); 2 S.C. Apel Laser S.R.L., 25 Vanatorilor Street, 077135 Ilfov, Romania; alin.enache@apellaser.ro; 3 Department of Public Health, Faculty of Veterinary Medicine “Ion Ionescu de la Brad”, University of Life Sciences, 8 Mihail Sadoveanu Alley, 707027 Iasi, Romania; crimbu@yahoo.com; 4 National Institute of Research and Development for Biological Sciences, 296 Independentei Bd. District 6, 060031 Bucharest, Romania; truicageorgiana@yahoo.com

**Keywords:** chitosan membranes, antimicrobial resistance, *S. officinalis*, *H. perforatum*

## Abstract

The main strategy of this study was to combine the traditional perspective of using medicinal extracts with polymeric scaffolds manufactured by an engineering approach to fabricate a potential dressing product with antimicrobial properties. Thus, chitosan-based membranes containing *S. officinalis* and *H. perforatum* extracts were developed and their suitability as novel dressing materials was investigated. The morphology of the chitosan-based films was assessed by scanning electron microscopy (SEM) and the chemical structure characterization was performed via Fourier transform infrared spectroscopy (FTIR). The addition of the plant extracts increased the sorption capacity of the studied fluids, mainly at the membrane with *S. officinalis* extract. The membranes with 4% chitosan embedded with both plant extracts maintained their integrity after being immersed for 14 days in incubation media, especially in PBS. The antibacterial activities were determined by the modified Kirby–Bauer disk diffusion method for Gram-positive (*S. aureus* ATCC 25923, *MRSA* ATCC 43300) and Gram-negative (*E. coli* ATCC 25922, *P. aeruginosa* ATCC 27853) microorganisms. The antibacterial property was enhanced by incorporating the plant extracts into chitosan films. The outcome of the study reveals that the obtained chitosan-based membranes are promising candidates to be used as a wound dressing due to their good physico-chemical and antimicrobial properties.

## 1. Introduction

Antimicrobial resistance, one of the major challenges of the 21st century, is the leading cause of mortality worldwide, with the greatest burdens in low-resource regions, as indicated by a comprehensive study that evaluates and estimates the data available up to 2019 [1]. According to this report, the six leading pathogens associated with antimicrobial resistance deaths (*E. coli*, *S. aureus*, *K. pneumoniae*, *S. pneumoniae*, *A. baumannii* and *P. aeruginosa*) were responsible for 929,000 deaths attributable to antimicrobial resistance and 3.57 million deaths associated with this in 2019. Intervention strategies to address the challenge of antimicrobial resistance can be divided into several main categories, such as prevention through some community-based control programs focused on water, sanitation or hygiene, prevention and control of infections through vaccination, reduction in exposure to antibiotics, minimizing the use of antibiotics or development of new antibiotics [2,3,4]. Many researchers have recently focused on herbal-based treatment methods to treat a variety of human health problems due to the broad pharmacological importance of medicinal plants [5]. On the basis of the available scientific information, plant extracts and oils have been extensively studied as effective antimicrobial and anti-inflammatory therapeutics [6,7,8,9]. Hence, plant-derived antimicrobials have a wide range of activity depending on the species, topography and climate of the country of origin and may contain various categories of active principles, such as polyphenols, quinones, flavonoids, tannins, terpenoids, alkaloids, etc. [10].

*Salvia officinalis*, also known as sage, is an ornamental, culinary, aromatic and medicinal plant that is mostly used in the pharmaceutical, perfumery and food industries [11]. Although this plant is frequently used as a condiment, its medical uses have opened the way for a new and promising research line. Thus, the essential oil or extract has shown various biological activities, including antioxidant [12,13], antibacterial [14,15,16], antiviral [17,18] and antifungal [19,20] properties, and also antitumor or antimutagenic effects [21]. It is widely used in the treatment of various diseases, such as those of the nervous system, heart and blood circulation, respiratory system, digestive system, metabolic, and endocrine disease [22]. All of these bioactivities are due to the major phytochemicals, such as alkaloids, phenolic compounds, glycosidic derivatives, terpenes, etc. [23,24].

*Hypericum perforatum* is a herbaceous perennial plant known also as St. John’s wort, which possesses a wide range of biological activities, such as antidepressant [25,26], antianxiety [27], anti-inflammatory [28], antimicrobial [29], wound healing [30] and anti-tumoral [31]. The biological activities are also due to the numerous phytochemicals that are included in the extracts, such as hypericin, pseudohypericin, rutin, quercetin, quercitrin, etc., leading to unique and combined medicinal effects [32]. Variations in these component contents in the plant are mainly related to the harvesting period, drying process and storage. Thus, due to its rich content of flavonoids, *H. perforatum* also presents antioxidant and free radical scavenger activity [33,34]. Other studies have shown its antimicrobial activity against a variety of bacterial and fungal strains [35,36]. Therefore, several phytochemicals found in the *H. perforatum* extract have been shown to support the desired pharmaceutical effect.

Chitosan is a natural carbohydrate polymer derived from the deacetylation of chitin through chemical or biological processes, which is found as a major component in the shells of crustaceans, such as crabs, shrimp and crawfish [37,38]. It is a cationic polysaccharide that exhibits a wide spectrum of antibacterial and antifungal activity against many bacteria, fungi and yeasts [39,40]. Moreover, due to its high biodegradability, biocompatibility, nontoxicity and similarity in structure to the extracellular matrix component, chitosan is widely used alone or blended with other natural or synthetic polymers in the food, pharmaceutical, medicine, agriculture, cosmetic industries, textiles, etc. [41]. Chitosan can be processed into different forms, such as nanoparticles, gel, membranes, nanofibers, beads and scaffolds, and it is widely applied in biomedical purposes, including drug delivery, regenerative therapy, tissue engineering, wound healing, cancer therapy, bioimaging or veterinary medicine [42,43]. The development of hydrogels based on chitosan is a useful tool for a variety of biomedical applications due to its high absorption capacity, ease of shaping, great capacity to produce a moist environment and ease of drug diffusion [44,45]. Several studies have found that chitosan alone has significant antimicrobial properties against various types of bacteria, including *S. aureus*, *L. plantarum*, *E. coli*, *Salmonella Enteritidis*, etc. [46,47]. Its antimicrobial activity is still unclear due to the complexity of its action on microorganisms that is not well understood, so there are several hypotheses regarding this aspect. One of the most accepted assumptions describes the electrostatic interactions between the positively charged amino groups of chitosan and the negatively charged microbial cell membrane, inducing changes in their permeability, leading to the inhibition of microbial growth [38,48,49]. Another assumption is the chelation of chitosan molecules with some metal ions, which damages the microorganism cell walls [46,50,51]. In addition, the effectiveness of the antimicrobial activity of chitosan is also dependent on its molecular weight. Therefore, low-molecular-weight chitosan is generally able to penetrate the cell wall, affecting DNA transcription [48]. Furthermore, the antimicrobial action of chitosan depends strongly on the type of microorganism. Thus, differences in the cell surface structure of the Gram-positive and Gram-negative bacteria lead to different sensitivity to chitosan. For example, more negatively charged cell surfaces of Gram-negative bacteria allow the binding of cationic chitosan to their phospholipids at low pH [47,48]. Taking into consideration these aspects, we used low-molecular-weight chitosan to prepare the membranes to increase both their extracellular and intracellular antimicrobial activity.

In recent years, researchers have become more and more interested in the synergistic effects of chitosan and various herbal extracts due to the broad pharmacological importance of medicinal plants and growing interest in alternative nanomedicine. Different scaffolds made of chitosan and various herbal extracts have been studied over the years, especially for wound dressing applications. Therefore, the possibility of incorporating natural extracts, such as *S. officinalis* extract, to enhance the antibacterial properties of chitosan has been explored, and a wide range of formulations was prepared. Thus, the *S. officinalis* extract was incorporated into chitosan films, hydrogels or nanoparticles in different forms: encapsulated in nano-form using niosome to enhance biodistribution and bioavailability [47], entrapped into an enzymatic biosensor based on multiwall carbon nanotubes–chitosan–laccase film for total polyphenolic content assessment [48], inserted into a chitosan-based hydrogel formulation for dermal applications [49] or entrapped in chitosan nanoparticles as a stable and protective vehicle to deliver rosmarinic acid [50]. *H. perforatum* extract or oil has also been used to prepare various chitosan-based matrices for wound healing [51,52,53], as a drug carrier [54] or in tissue engineering applications [55]. Thereby, the encapsulation of the plant extracts in chitosan-based carriers facilitates their delivery in a regulated manner, targeting therapeutic action, and the nanoscale dosage reduces side effects during medical treatment. Therefore, the aim of our study was to develop a novel scaffold by combining the antimicrobial properties of *S. officinalis* and *H. perforatum* extracts with the unique properties of low-molecular-weight chitosan and to investigate its suitability as a potential biomaterial used in tissue engineering or in wound healing applications. The main strategy was to develop a soft and stable chitosan-based scaffold embedded with plant extracts manufactured by an engineering approach. Moreover, we want to underline the potential application of these two extracts for biomedical formulations considering their natural benefits.

## 2. Results and Discussion

### 2.1. HPLC Characterization of Plant Extracts

Generally, the chemical composition of plant extracts varies depending on their geographic origin, botanical sources, storage time and conditions, extraction techniques and parts of the plants used during extraction (fruit, stems, leaves, roots or seeds), increasing the difficulty in comparing the modes of action of the main active compounds [8,23,52,53]. Considering these aspects, HPLC analysis was performed in order to identify and quantify the compounds found in the extracts based on the retention time and similarity of UV spectra with the standard substances.

The main constituents of *S. officinalis* extract and *H. perforatum* powder used in the experiments are presented in Table 1*. Salvia* and *Hypericum* species are known to be rich in phenolic acids and flavonoids. Thus, in the obtained *S. officinalis* extract was reported the quantitative determination of flavonoids, including catechin, naringenin, hesperidin, apigenin, pinocembrin, rutin, quercetin, myricetin, rhamnetin or kaempferol, in addition to the different phenolic acids, such as gallic acid, ellagic acid, caffeic acid, caftaric acid, p-coumaric acid, ferulic acid or rosmarinic acid. The presence of these compounds was detected in many species of *S. Officinalis* extracts [21,54,55,56,57] at different concentration levels. Numerous flavonoid compounds, such as isoquercitrin, rutin, epicatechin, kaempferol or myricetin, are found in the aboveground portions of the *H. perforatum* plant. Moreover, different phenolic acids (ellagic acid, caffeic acid, caftaric acid, p-coumaric acid or ferulic acid) appeared in both studied plants [57,58]. Actually, the synergistic effects of the variety of minor and major constituents of the studied plant extracts should be considered for their biological activity.

### 2.2. ATR-FTIR Characterization of Chitosan and Chitosan Plant Extract Membranes

**Figure 1:**
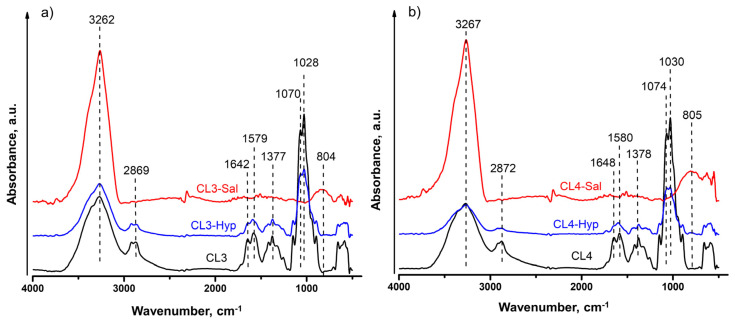
ATR-FTIR spectra of (**a**) 3 %wt and (**b**) 4 %wt pure chitosan and chitosan plant extract membranes.

ATR-FTIR microscopy was used to determine the chemical binding and functional groups of the chitosan membranes and chitosan plant extract membranes. The recorded spectra of chitosan plant extract membranes were compared to those of chitosan membranes, as illustrated in Figure 1. Firstly, we analyzed the ATR-FTIR spectra of both CL3 and CL4 pure chitosan membranes, which presented a similar pattern. Thus, the pure chitosan membranes’ spectra showed a broad absorption peak between 3600 cm^−1^ and 3100 cm^−1^ that can be attributed to O–H and N–H stretching vibrations. The peaks corresponding to asymmetric and symmetric stretching vibrations of C–H bonds in -CH_2_ and -CH_3_ groups appeared at 2920 and 2870 cm^−1^, respectively. The absorption band between 1700 cm^−1^ and 1500 cm^−1^ was related to the vibrations of C=O bonds of amide groups (amide II) (1645 cm^−1^) and to the vibrations of amine groups (1580 cm^−1^). Bending vibrations of methyl and methylene groups were also visible at around 1416 cm^−1^ and 1377 cm^−1^, respectively. The spectra between 1160 cm^−1^ and 1000 cm^−1^ are attributed to the vibrations of the CO groups. Thus, according to literature data [59], the small peak located around 1145 cm^−1^ could be attributed to the asymmetric vibrations of CO in the oxygen bridge, resulting from the deacetylation of chitosan. Then, the peaks around 1075–1030 cm^−1^ are attributed to the vibration of CO groups of the ring COH, COC and CH_2_OH.

Then, we investigated the IR spectra of chitosan membranes embedded with plant extracts, which clearly showed a different profile when compared to that of pure chitosan membranes. This is mainly due to the fact that the composition of the plant extracts is quite complex and dissimilar (Supporting Information Figure S1) and thus the absorption peaks characteristic of the chitosan structure overlap with those of the plant extract. Despite these inconveniences, some general conclusions could still be drawn. Therefore, analysis of the chitosan plant extract membranes showed that the profile of all the spectra changed after plant extract loading because of the intermolecular rearrangement and variations in the configuration of the main chain. Moreover, these changes are also due to the synergistic effect of phenolic compounds in the extracts and the interactions between them and the chitosan chain structure [60]. As a result, the broad absorption bands in the region between 3600 cm^−1^ and 3100 cm^−1^ corresponding to the -OH stretching vibrations have increased in intensity when compared to the spectrum of pure chitosan membranes. On this basis, it can be assumed that the chitosan molecules have established intra- and intermolecular hydrogen bonds through their -OH and -NH functional groups with -OH groups of the most prevalent components from the plant extracts, as proposed in the study of Bajic et al. [61].

### 2.3. SEM Evaluation of Chitosan and Chitosan Plant Extract Membranes

**Figure 2:**
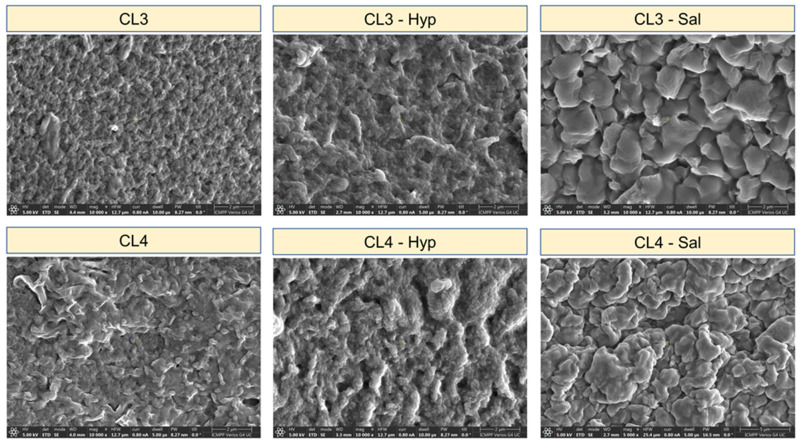
SEM micrograph of pure chitosan and chitosan plant extract membranes.

The SEM analysis was performed in order to visualize the surfaces of chitosan-based membranes before and after the addition of the plant extracts (Figure 2). This analysis can directly provide information on the morphology of the studied sample surfaces. Thus, the SEM micrographs revealed that all the samples presented ridge-and-valley structures with continuous and compact surfaces, without any visible cracks or holes. However, some variations in the surface morphology of the membranes were observed depending on the composition. The plant extract’s incorporation changes the surface morphology, leading to rough surfaces after their addition as a result of the rise in height of the ridge-and-valley structures. This phenomenon may be due to the spatial organization and the interactions of the constituents during the preparation of the membranes.

### 2.4. In Vitro Incubation

#### 2.4.1. Swelling Capacity of Membranes

**Figure 3:**
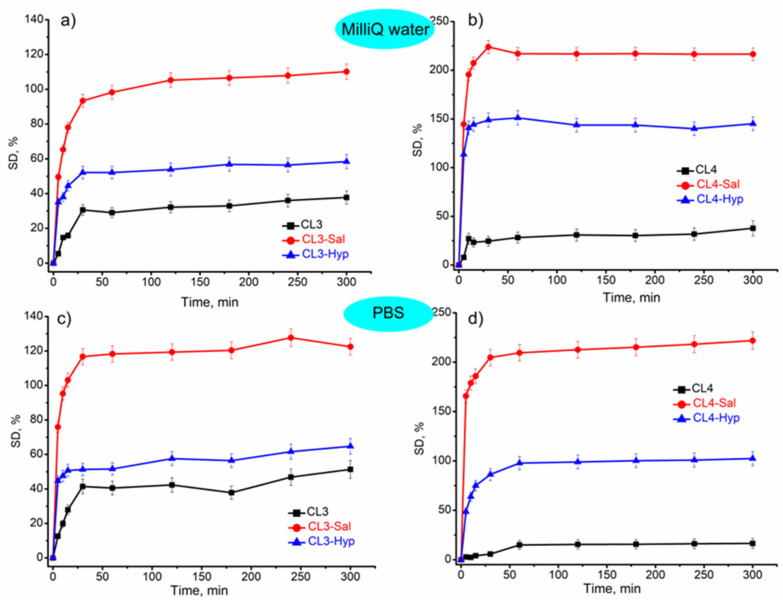
Swelling of membranes with or without plant extracts in (**a**,**b**) Milli-Q water and (**c**,**d**) PBS medium. The values are represented as mean ± standard deviation of triplicate experiments.

The water/fluid uptake capacity of the samples according to their plant extract composition was evaluated in Milli-Q water and PBS medium at 37 °C at specific intervals. The water uptake capacity/swelling of the investigated samples is presented in Figure 3. The swelling capacity of all the samples increased as time increased and reached equilibrium after around 30 min, maintaining their structural integrity for 300 min. After this time, the experiment was stopped because the samples began to deteriorate due to weight loss. The equilibrium swelling of hydrogels is the result of the balance of osmotic forces determined by the affinity for the solvent as well as by the elasticity of the network. Thus, the investigated membranes presented a sharp increase in water uptake in the first half hour and then began to level off. The sorption capacity of the samples with 4% chitosan (up to 200%) was higher than the samples with 3% chitosan (up to 100%). The PBS medium does not have a great influence on the sorption capacity when compared with pure water. The swelling degree profile in both media is almost the same. The samples without plant extract have the lowest swelling degree, while those with *S. officinalis* extract showed the highest swelling capacity. This trend was observed in all the samples. These results suggested that the addition of plant extracts led to an increase in the sorption capacity of fluids. This could be the result of fluids penetrating the spaces that plant extract had previously occupied inside the polymer network after it was released, leading to higher swelling ratios. The analogous results were presented by Drabczyk et al., who used *Aloe vera* embedded in hydrogel materials [62].

The swelling or water uptake capacity of a hydrogel is an important property, especially in wound healing when exudate absorption is required for healing [63]. Therefore, it is essential to develop some new materials with optimal sorption properties adequate for their use and to provide the optimal environment for wound healing.

#### 2.4.2. PH Metric Analysis

**Figure 4:**
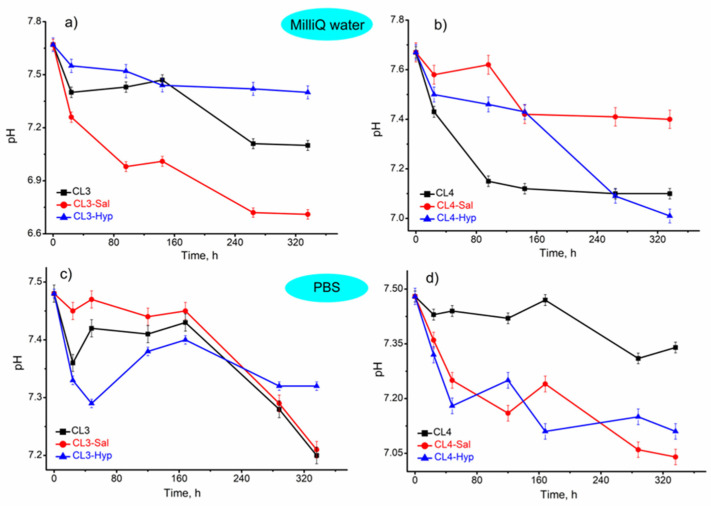
pH stability of membranes with or without plant extracts in (**a**,**b**) Milli-Q water and (**c**,**d**) PBS medium. The values are represented as mean ± standard deviation of triplicate experiments.

The influence of the membranes with or without plant extracts on the immersed fluids was also evaluated. This incubation study was conducted in order to observe the changes and perhaps the interactions that could appear when the membranes were soaked for 2 weeks in PBS and water. The results were illustrated in Figure 4. According to these results, it was observed that the pH decreases over time in both media. The samples immersed in Milli-Q water presented a decrease in the initial pH from 7.67 to 6.71 (CL3-Sal). The same trend was observed in PBS medium, when the pH decreased from 7.48 (initially) to 7.04 (CL4-Sal).

This decrease in pH is most probably due to the synergy of two factors. One of them is the release of the plant extracts in the incubated media since the initial pH of the plant extract solutions in ethanol was 6.79 and 6.48 for Sal and Hyp, respectively. Another could be the release of some molecules or ions from the membranes, mostly due to their partial degradation. Thus, the tested membranes had a small effect on the pH of the tested fluids.

Moreover, in Milli-Q water, the decrease in pH is more stable than in PBS medium, where fluctuation in the values was noted. This could be explained by the difference in the tested fluids. In the case of Milli-Q water, there were no free ions in the incubation medium that could interact with the tested membranes compared to PBS. The ions from the PBS medium could interact with the samples, leading to some variation in the values. Since the decrease in pH is around 7.0, we could draw the conclusion that the investigated membranes can be considered compatible with the selected fluids.

#### 2.4.3. Mass Loss of Membranes

**Figure 5:**
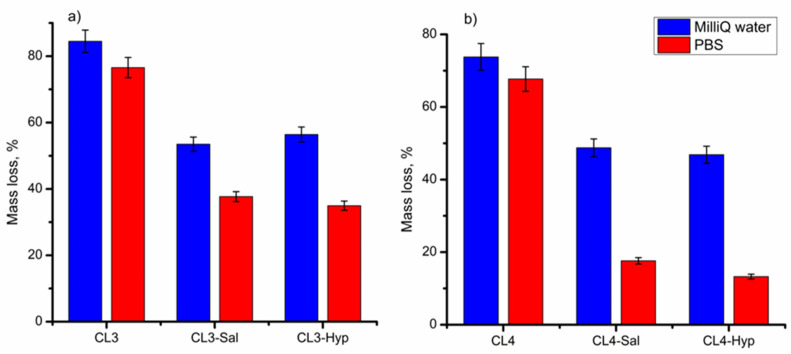
Mass loss after 14 days of incubation in Milli-Q water and PBS for (**a**) 3 %wt and (**b**) 4 %wt membranes with or without plant extracts. The values are represented as mean ± standard deviation of triplicate experiments.

The loss in the weight of the investigated membranes with or without plant extracts was evaluated in Milli-Q water and PBS medium after 14 days of incubation. The mass loss was calculated using equation 2 and the results are illustrated in Figure 5. As observed, the degradation rate of the pure chitosan membranes (CL3 and CL4) was higher than the membranes with plant extracts. Thus, the mass loss of the CL3 and CL4 samples after incubation in water was around 84% and 74%, and in PBS was about 76% and 67%, respectively. The addition of the plant extracts led to the decrease in the membranes’ mass loss up to 13% for the CL4-Hyp in PBS. This may be because the plant extracts could act as a crosslinker, which diminishes the hydrolytic degradation of the membranes. When comparing the mass loss in the two incubation media (Milli-Q water and PBS), it was observed that the membranes incubated in PBS were more stable, resulting in less mass loss. This is due to the presence of the ions in the PBS media, which led to a more packed structure with lower liquid absorption capacity [64].

In conclusion, the chitosan membranes with a concentration of 4% embedded with both plant extracts maintained their integrity after being immersed for 14 days in incubation media, especially in PBS. Thus, these membranes are suitable for applications that require higher stability, such as a wound dressing, which should remain in contact with the skin for a long period.

### 2.5. Antimicrobial Properties of Active Chitosan Membranes

As a key parameter, the antimicrobial property assessment is necessary for evaluating the eligibility and capability of these membranes. Mediterranean medicinal and aromatic herbs contain a wide range of bioactive compounds (polyphenols, terpenes, etc.) that are important constituents of herbal extracts. *S. officinalis* and *H. perforatum* extracts were chosen to be incorporated into the chitosan membranes due to their antimicrobial properties recognized in several works [14,15,16,29,35,36]. The antimicrobial capacity and potential health benefits of these bioactives could be associated with their synergistic effects.

The antimicrobial activity of the *H. perforatum* and *S. officinalis* extracts, as well as chitosan membranes loaded with both extracts, was tested in triplicate using the diffusimetric technique. The results were interpreted on the basis of the geometric mean of the values obtained by measuring the diameter of the inhibition zones. To highlight the accuracy of the obtained results, the standard deviation from the mean (mean ± SD) was also calculated (Table 2). Considering the differences in bacterial structure and behavior towards antimicrobial agents, the tests were performed against Gram-positive (*Staphylococcus aureus* ATCC 25923, *Methicillin-resistant Staphylococcus aureus* ATCC 43300) and Gram-negative (*Escherichia coli* ATCC 25922, *Pseudomonas aeruginosa* ATCC 27853) bacterial species.

**Figure 6:**
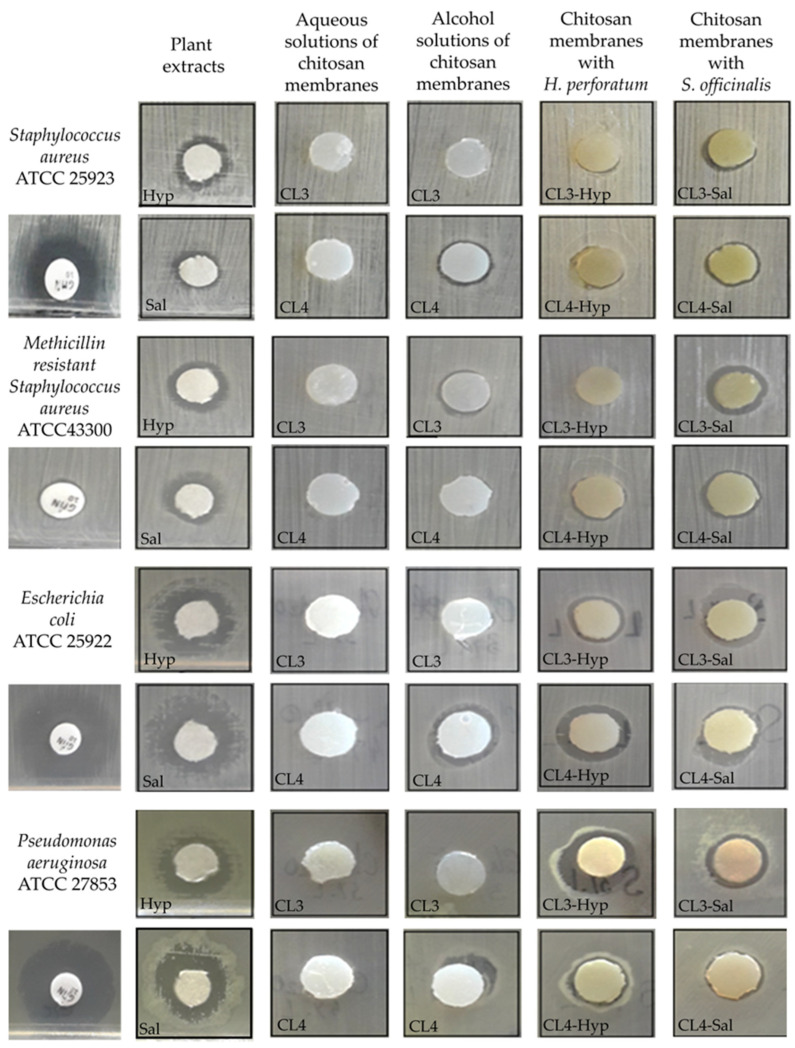
Illustration of the antimicrobial effect tested by diffusimetric method of the investigated samples using Gram-positive and Gram-negative bacteria.

**Figure 7:**
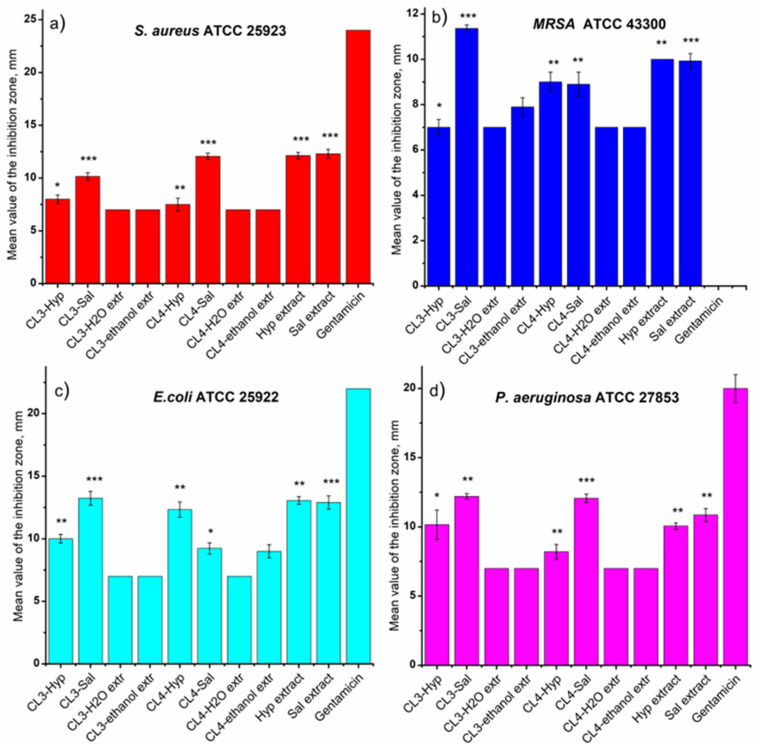
The antimicrobial activity of the investigated samples against Gram-positive (**a**) *S. aureus* and (**b**) *MRSA* and Gram-negative (**c**) *E. coli* and (**d**) *P. aeruginosa* bacteria. The values are represented as mean ± standard deviation of triplicate experiments. Statistical significance: *** *p* < 0.001; ** 0.001 < *p* < 0.01; * 0.01 < *p* < 0.05.

The antimicrobial activity of the chitosan membranes was influenced by the type of the plant extracts (Table 2, Figure 6). The results show that the extract of *S. officinalis* incorporated into the chitosan membranes has slightly stronger antimicrobial activity against Gram-positive bacteria than the chitosan membranes loaded with *H. perforatum* (Figure 6). In this regard, it was found that CL3-Hyp (8 mm) and CL4-Sal (12.06 mm) samples inhibited the multiplication of *S. aureus* species (Figure 7a). Against *MRSA* species (Figure 7b), the antimicrobial potential reversed so that the CL4-Hyp (9 mm) and CL3-Sal (11.36 mm) samples were most effective. This aspect is also maintained when Gram-negative species are tested, the extract of *S. officinalis* from chitosan membranes being more active than the extract of *H. perforatum* (Table 2). The exception is the CL4-Sal (9.23 mm) membrane, which was tested against *E. coli* (Figure 7c). Apparently, both chitosan membranes loaded with *S. officinalis* extract have almost identical inhibitory activity against *P. aeruginosa* (CL3-Sal, 12.2 mm and CL4-Sal, 12.06 mm), (Figure 7d).

The tests of *S. officinalis* and *H. perforatum* extracts (10 µL) highlighted the antimicrobial potential of both plant extracts. The ranges of microbial inhibition obtained against *S. aureus* (*H. perforatum*—12.13 mm and *S. officinalis*—12.3 mm), *MRSA* (*H. perforatum*—10 mm and *S. officinalis*—9.93 mm), *E. coli* (*H. perforatum*—13.06 mm and *S. officinalis*—12.9 mm) and *P. aeruginosa* (*H. perforatum*—10.06 mm and *S. officinalis*—10.86 mm) are close in their values (Table 2 and Figure 6 and Figure 7).

*H. perforatum*, the best-known species of the genus *Hypericum,* is widely used in traditional medicine for its sedative, analgesic, anthelmintic, anti-inflammatory, and antimicrobial properties [65,66]. The literature data frequently refer to the bactericidal potential of both extracts and essential oils of *T. perforatum*, which are mainly effective against Gram-positive bacteria [65,67,68], but not exclusively [69]. In our study, the pure extract of *H. perforatum* or embedded in chitosan membranes induced very similar antimicrobial activity against Gram-negative (*E. coli* and *P. aeruginosa*) and Gram-positive (*S. aureus*, *MRSA*) species. Such results were also mentioned by other authors [29]. It is known that the antimicrobial activity of plant extracts is the cumulative effect of polyphenols (flavonoids or hydrolysable tannins) containing one or more phenolic groups [70]. The most known active compounds of *H. perforatum* that exhibit antimicrobial activity include hypericin, pseudohypericin, naphthodianthrones, flavonoids, phloroglucinols, hyperforin and adhiperforin [66,71]. The main compounds identified in the extract from our study were isoquercitin, epicatechin, rutin and ellagic acid. These compounds are known to have in vitro antimicrobial properties [72,73], especially when they are combined with other identified molecules, attributing real biological properties to the *H. perforatum* extract.

The ethanolic extract of *S. officinalis* is known to have potent antifungal [74] and antibacterial properties against both Gram-positive and Gram-negative bacteria [75,76]. Its antimicrobial effects are closely related to the origin of the plants, the time of harvest and other factors. Sage essential oils are rich in thujone, camphor and 1,8-cineole [77], while camphor predominates in hydrolats [76]. They are considered the most important compounds for antibacterial activity [78]. The pure extract of *S. officinalis* embedded in chitosan membranes inhibited bacterial proliferation of both Gram-positive and Gram-negative species, with a slightly stronger antimicrobial effect than that of *H. perforatum*. These differences in the fluctuation of the antimicrobial activity were mainly due to the different composition and concentration of polyphenols.

In both extracts, isoquercetin was the major flavonoid, whose mechanism of antimicrobial action is based on lipid peroxidation in both Gram-negative and Gram-positive bacteria in response to oxidative stress, leading to membrane liquefaction and permeabilization, and ultimately apoptosis [73]. 

It is also known that positively charged amino groups of chitosan can electrostatically interact with negatively charged components on the surface of the microbial cell, leading to the damage of membranes’ cell walls. Thus, the protonated groups of chitosan are bonded to the negatively charged teichoic acid of peptidoglycans, resulting in disruption of bacterial cell membranes, altering the cell permeability and barrier properties [38,46]. The death of the cells is due to the loss of essential cell materials, such as proteinaceous and other intracellular constituents [40]. This hypothesis is considered to be the widely accepted mechanism of antimicrobial activity of chitosan against Gram-positive and Gram-negative bacteria [47,48]. Furthermore, the chitosan with low molecular weight has the ability to penetrate through the cell wall structure into the cytoplasm of the bacteria, altering the DNA/RNA activity and ultimately inducing cell death [46]. This is because small chains exhibit easier mobility, attraction and ionic interactions than larger ones. In general, the lower the molecular weight, the higher will be the effectiveness in inhibiting microorganisms’ growth and multiplication [79]. This is another proposed mechanism of antimicrobial action that is accepted in the literature.

The developed biomaterials based on chitosan embedded with both extracts of *H. perforatum* and *S. officinalis* can be used in many medical fields or textile technology due to their special antimicrobial properties. Considering the performances of the investigated chitosan-based membranes embedded with plant extracts and initiating a path toward the clinical implementation of such biomaterials, further investigations need to be carried out in future studies.

## 3. Materials and Methods

### 3.1. Materials

Low-molecular-weight chitosan with a degree of deacetylation of 75–85% and an average molecular weight of 50–190 kDa (based on viscosity), *Hypericum perforatum* extract (hypericin 0.3 mg/g), acetic acid (glacial, >99%) and ethanol (absolute for analysis) were purchased from Sigma-Aldrich (Taufkirchen, Germany). Sodium phosphate buffer solution (PBS), pH = 7.4, was prepared by following the standard protocol, using KH_2_PO_4_ (1.8 mM), Na_2_HPO_4_ (10 mM), NaCl (137 mM) and KCl (2.7 mM) dissolved in Milli-Q water. Milli-Q water (18.2 MW∙cm) was produced by an Integrity+ Ultrapure water purification system (Adrona, Riga, Latvia). All other chemicals and reagents were of analytical grade and used as received without further purification.

### 3.2. Plant Materials and Extraction

The extraction of *S. officinalis* was described in a previous study [80]. Briefly, in a mortar, the dried plant buds or leaves were grounded. They were put through a sieve with mesh sizes between 20 and 30, and particle diameters ranging from 0.60–0.85 mm, in order to obtain reproducible extraction yields. The obtained product was stored in a sealed bag in a cool, dry location until they were used [81]. Aldrich^®^ Soxhlet Extraction Equipment, Z556203 (St. Louis, MO, USA), was used for the extraction, which lasted 6 h. This extraction was carried out using 150 mL of ethanol and a known quantity (6 g) of dried materials. The temperature was maintained at 60 °C during the extraction. Then, the solvent was evaporated at 45 °C under reduced pressure in a rotary evaporator (Heidolph Instruments GmbH& CoKG, Schwabach, Germany) to obtain the crude ethanol extract. This was then stored at −20 °C.

### 3.3. Analysis of Polyphenols by HPLC-UV

In order to identify and characterize the phenolic compounds, a sample of 10 μL of the extracts was analyzed using a Shimadzu LC-20AT HPLC system equipped with a quaternary pump, solvent degasser, autosampler and a photodiode (PDA) detector. The separation of the compounds was performed on a 150 × 4.6 mm, 5 μm Kinetex C18 column, (Agilent Technologies, Santa Clara, CA, USA) using mobile phases A (water and phosphoric acid, pH 2.3) and B (acetonitrile) in a gradient mode (between 5 and 90% component B) for 45 min at a temperature of 35 °C, with a flow rate of 0.8 mL/min. The components of the mobile phase were filtered and degassed before starting the HPLC daily run, through Macherey-Nagel (MV) filters, 0.20 μm pore size and using an Elma-Elmasonic P sonication bath. Detection was performed by scanning from 190 to 800 nm and quantification was assessed at three specific wavelengths (280, 320 and 360 nm). The acquisition of data and the interpretation of the results occurred using the LC Solution software. Concentrations of standard compounds in extracts were determined from the peak areas using the equation for linear regression obtained from the calibration curves.

### 3.4. Preparation of Chitosan Membranes

In this study, we used low-molecular-weight chitosan for the preparation of the membranes due to its enhanced antimicrobial activity. Thus, the chitosan hydrogels were obtained as previously reported [82] by inducing the gelation of the chitosan solution with aqueous NaOH. Firstly, two chitosan solutions (3%, 4%wt.) were prepared by adding a stoichiometric amount of acetic acid in order to protonate all the amino groups of the chitosan molecules. The chitosan solutions were then placed in Petri dishes (D = 40 mm), immersed in a Berzelius glass containing 1 M NaOH aqueous solution and left a predetermined time to obtain the chitosan membranes. After the desired coagulation time, the Petri dishes were taken out from the NaOH solution. The uncoagulated chitosan solution was removed, and the chitosan membranes were placed into a Berzelius glass with deionized water in order to wash out the NaOH. The washing was carried out until the pH of the chitosan membranes supernatant was neutral.

### 3.5. Preparation of Chitosan-Based Membranes Containing Plant Extracts

The membranes containing plant extracts were prepared by absorption of plant solutions. Thus, 0.1% stock solutions of plant extracts were freshly prepared in ethanol. Each solution was sonicated for 10 min to ensure a homogenous solution. The chitosan membranes were cut into suitable sizes (Ø = 2 cm) according to the dimensions of the glass vials. Then, 1 mL from the plant extracts stock solution was added and the vials were shaken at 37 °C for 48 h until the solution was completely absorbed by the membranes. The resulting membranes were then freeze-dried using an ALPHA 1–2 LD Christ lyophilizer (Osterode, Germany). The samples were denoted as CLx, CLx-Sal and CLx-Hyp, respectively, where x represents the concentration of low-molecular-weight chitosan (3 and 4 %wt.)

### 3.6. Characterization of Chitosan-Based Membranes

Attenuated total reflection Fourier transform infrared (ATR-FTIR) spectra of the chitosan-based membranes with or without plant extracts were recorded using a Bruker LUMOS FTIR microscope spectrometer (Bruker Corporation, Karlsruhe, Germany) equipped with an ATR reflection module (Attenuated Total Reflection) and a diamond crystal. The previously prepared samples were cut into small pieces, placed directly on the ATR crystal and subjected to IR investigation. All the spectra were collected in the range 500–4000 cm^−1^, and the measurements were made by averaging 64 scans at a resolution of 2 cm^−1^. The spectra were recorded at room temperature.

The surface morphology of the chitosan-based membranes with or without plant extracts was studied by scanning electron microscopy (SEM). The samples were cut into small pieces, mounted on a stub, coated with a thin layer of platinum in a sputtering device and then examined on a Verios G4 UC scanning electron microscope (Thermo Scientific, Waltham, MA, USA) equipped with an energy-dispersive X-ray spectroscopy analyzer (Octane Elect Super SDD detector (AMETEK, Tokyo, Japan)).

### 3.7. In Vitro Incubation

#### 3.7.1. Swelling Capacity of Membranes

The swelling ability of membranes was performed by conventional gravimetric procedure to calculate the water uptake capacity and equilibrium state of the samples with respect to time at body temperature (37 °C). Thus, the swelling degree (%SD) of chitosan-based membranes with or without plant extracts was determined in PBS and Milli-Q water. The membranes were cut in 1 × 1 cm dimensions, weighed and placed in closed glass containers each containing 10 mL of liquids. Then, the samples were incubated at 37 °C in a thermostated oven. At the predetermined time, the membranes were weighed after removing the water excess from the surface with filter paper. The swelling degree (%SD) of the membranes was calculated according to the following equation:

%SD = (W − Wo)/Wo ∗ 100(1)

where W and Wo represent the weight of the membranes in the wet and dry states. The results were provided as mean ± standard deviation.

#### 3.7.2. PH Metric Analysis

To evaluate the influence of the membranes on the incubation fluids in vitro, a pH metric study was performed. Our purpose was to observe the pH changes in the fluids used to incubate the investigated membranes over time. The change in the pH values is due to the interaction between the samples and the incubation fluids. Thus, membrane pieces were placed in closed glass containers containing phosphate buffer saline (PBS) and Milli-Q water and incubated at 37 °C. All samples were prepared in triplicate, and the pH was recorded after measurement stabilization, for two weeks, using a pH meter (Hanna Instruments, Woonsocket, RI, USA).

#### 3.7.3. Mass Loss of Membranes

The mass loss was achieved by immersing the pre-weighed membrane pieces (1 × 1 cm) with or without plant extract in tightly closed glass containers containing 10 mL solutions of phosphate buffer saline (PBS) or Milli-Q water. The samples were kept at 50 rpm and 37 °C in a shaker incubator for 14 days. After this interval of time, the samples were rinsed with Milli-Q water and dried in an oven for 48 h. The degradation process was monitored by measuring the weight changes after 14 days of incubation. The percentage of weight loss was calculated using the following equation:

% W = (Wi − Wt)/Wi ∗ 100(2)

where Wi and Wt are the weights before and after incubation in solutions.

### 3.8. Evaluation of Antimicrobial Activity of Chitosan-Based Membranes

The antibacterial properties of the pure plant extracts and embedded in chitosan membranes were evaluated using the modified Kirby–Bauer disk diffusion method [83]. The aqueous solutions (CL3 + H_2_O, CL4 + H_2_O) and the alcoholic solutions (CL3 + ethanol, CL4 + ethanol) served as controls for chitosan membranes without plant extracts. The extracts of *H. perforatum* (Hyp), *S. officinalis* (Sal) and a standardized antibiotic disk (gentamicin, 10 µg, Oxoid) served as controls for the evaluation of antimicrobial activity. Antimicrobial activity was tested against Gram-positive (*Staphylococcus aureus* ATCC 25923, *Methicillin-resistant Staphylococcus aureus* ATCC 43300) and Gram-negative (*Escherichia coli* ATCC 25922, *Pseudomonas aeruginosa* ATCC 27853) bacteria. Cell suspensions were prepared from the 24 h bacterial cultures at a density corresponding to a turbidity of 0.5 on the McFarland scale (1.5 × 10^8^ bacterial cells/mL). Sterile Mueller–Hinton agar (Oxoid) was added to sterile Petri plates, melted and cooled to 45 °C, whereupon 1 mL of the bacterial suspension was added. After drying, usually for 5 min on the thermostat (37 °C), sterile filter paper disks (Ø 7 mm) were spread on the surface of the medium, onto which 10 µL of all test solutions was spotted. Plates were prepared and incubated at 37 °C for 24 h.

### 3.9. Statistical Analysis

The presented data correspond to the mean ± standard deviation of three experimental values. The statistical differences between data were evaluated by one-way analysis of variance (ANOVA), and *p* < 0.05 was considered to be statistically significant.

## 4. Conclusions

In this study, chitosan membranes with two different plant extracts were prepared and investigated in terms of antibacterial activity. Commercial *H. perforatum* and *S. officinalis* extracts were used for the preparation of membranes in order to determine their potential use as biomaterials for infection healing. The quantitative analysis of flavonoids and different phenolic acids of the used plant extracts was carried out by HPLC method. The ATR-FTIR spectra demonstrated the successful incorporation of both plant extracts in the chitosan matrix by the presence of the characteristic components.

The swelling degree of the investigated membranes was influenced by the addition of the plant extracts, mainly at the membrane with *S. officinalis* extract. The chitosan-based membranes with or without plant extracts had a slight impact on the pH of the incubation media (PBS and Milli-Q water).

A small decrease in the pH value was noticed over time in both cases. This can be attributed to the release of the plant extracts in the incubated solutions on the one hand and the release of some molecules or ions from the membranes on the other hand, mainly due to their partial degradation. The degradation rate of the pure chitosan membranes (CL3 and CL4) was higher than the membranes with plant extracts after 14 days of incubation. The addition of the plant extracts led to a decrease in the membranes’ mass loss up to 13% for the CL4-Hyp in PBS. 

The plant extracts conferred significant antibacterial effects to the chitosan-based membranes toward *S. aureus* ATCC 25923, *MRSA* ATCC 43300, *E. coli* ATCC 25922 and *P. aeruginosa* ATCC 27853. The extract of *S. officinalis* incorporated into the chitosan membranes has slightly stronger antimicrobial activity against Gram-positive bacteria than the chitosan membranes loaded with *H. perforatum.*

Additionally, synthetic and chemical antibacterial agents that are known to be harmful to the environment and human health were avoided. Instead, composite polymeric membranes were created using common biomedical materials, such as chitosan and herbal extracts that are completely eco-friendly. It is possible to combine the special properties of polymeric materials with entirely natural herbal solutions to lead an innovative and environmentally friendly approach with potential use in various pharmaceutical or medical applications. This could be induced by the morphological and antibacterial properties of the composites, selecting the proper compositions of polymer-extract types. Thus, all the results demonstrated that these membranes are suitable for applications requiring higher stability, such as a wound dressing that should remain in contact with the skin for a long period.

## Figures and Tables

**Table 1:** HPLC analysis of *H. perforatum* powder and *S. officinalis* extract.

Nr. crt	Compound	*H. perforatum*		*S. officinalis*	
		t_R_ (min)	Conc (μg/mL)	t_R_ (min)	Conc (μg/mL)
1	Gallic acid	Nd	-	3.58 ± 0.01	subLOD
2	Catechin	Nd	-	7.62 ± 0.04	11.64 ± 1.57
3	Epicatechin	9.11 ± 0.03	90.61 ± 0.91	Nd	-
4	Ellagic acid	12.19 ± 0.003	71.92 ± 0.47	12.33 ± 0.01	38.22 ± 0.06
5	Hesperidin	14.33 ± 0.01	24.83 ± 1.34	14.52 ± 0.004	34.72 ± 1.17
6	Daidzein	Nd	-	Nd	-
7	Cinnamic acid	Nd	-	Nd	-
8	Apigenin	Nd	-	20.75 ± 0.01	21.88 ± 0.70
9	Genistein	21.20 ± 0.31	54.95 ± 0.59	Nd	-
10	Chrysin	Nd	-	27.29 ± 0.02	2.06 ± 0.29
11	Pinocembrin	27.68 ± 0.004	0.83 ± 0.09	27.62 ± 0.02	8.46 ± 0.32
12	Pinostrobin	Nd	-	Nd	-
13	Caftaric acid	6.36 ± 0.03	4.45 ± 0.31	6.62 ± 0.08	3.85 ± 0.12
14	Chlorogenic acid	7.99 ± 0.04	9.18 ± 1.69	Nd	-
15	Caffeic acid	9.11 ± 0.03	9.94 ± 0.05	8.85 ± 0.14	subLOD
16	Cynarine	9.91 ± 0.01	24.62 ± 0.01	10.00 ± 0.03	16.59 ± 0.05
17	P-Coumaric acid	11.63 ± 0.01	16.32 ± 0.04	11.64 ± 0.02	14.92 ± 0.16
18	Ferulic acid	12.62 ± 0.004	16.56 ± 0.21	12.81 ± 0.01	7.62 ± 0.37
19	Rosmarinic acid	Nd	-	14.93 ± 0.01	29.12 ± 0.10
20	Naringenin	Nd	-	21.30 ± 0.01	subLOD
21	Rutin	12.19 ± 0.003	105.97 ± 0.10	12.29 ± 0.01	1.79 ± 0.18
22	Isoquercetin	12.62 ± 0.004	183.95 ± 0.91	12.81 ± 0.01	124.18 ± 0.54
23	Myricetin	15.35 ± 0.03	9.13 ± 0.35	14.92 ± 0.01	34.36 ± 0.01
24	Quercetin	Nd	-	18.63 ± 0.01	9.83 ± 1.89
25	Kaempferol	21.25 ± 0.01	15.95 ± 0.12	21.61 ± 0.01	28.92 ± 0.29
26	Rhamnetin	24.41± 0.002	13.87 ± 0.23	25.06 ± 0.01	36.49 ± 0.41

**Table 2:** The mean value and standard deviation (SD) of the inhibition zones for the investigated samples obtained from the antimicrobial tests against different bacteria strains.

Samples	Initial Diameter	*S. aureus* ATCC 25923	*MRSA* ATCC 43300	*E. coli * ATCC 25922	*P. aeruginosa * ATCC 27853
	(Ø) (mm)	Mean ± SD Ø (mm)	Mean ± SD Ø (mm)	Mean ± SD Ø (mm)	Mean ± SD Ø (mm)
CL3-Hyp	7	8 ± 0.4	7 ± 0.34	10 ± 0.346	10.16 ± 1.06
CL4-Hyp	7	7.5 ± 0.60	9 ± 0.43	12.33 ± 0.61	8.2 ± 0.53
CL3-Sal	7	10.16 ± 0.35	11.36 ± 0.152	13.23 ± 0.55	12.2 ± 0.2
CL4-Sal	7	12.06 + 0.30	8.9 ± 0.53	9.23 ± 0.45	12.06 ± 0.30
CL3 + H_2_O	7	7 ± 0	7 ± 0	7 ± 0	7 ± 0
CL4 + H_2_O	7	7 ± 0	7 ± 0	7 ± 0	7 ± 0
CL3 + ethanol	7	7 ± 0	7.9 ± 0.41	7 ± 0	7 ± 0
CL4 + ethanol	7	7 ± 0	7 ± 0	9 ± 0.53	7 ± 0
*H. perforatum* Extract (10 ul) Control	7	12.13 ± 0.30	10 ± 0	13.06 ± 0.30	10.06 ± 0.23
*S. officinalis* Extract (10 ul) Control	7	12.3 ± 0.43	9.93 ± 0.32	12.9 ± 0.53	10.86 ± 0.47
Gentamicin (10 ug) Control	5	24 ± 0	0 ± 0	22 ± 0	20 ± 2

## Data Availability

Not applicable.

## Supplementary Materials

The following supporting information can be downloaded at: https://www.mdpi.com/article/10.3390/ijms24108673/s1.
