# Supplementary material for: Chitosan Membranes Containing Plant Extracts: Preparation, Characterization and Antimicrobial Properties
Source: Int J Mol Sci. 2023 May 12;24(10):8673. doi: 10.3390/ijms24108673 (PMC10217953; doi:10.3390/ijms24108673)
Supplement: Supplementary file 1 [file ijms-24-08673-s001.zip › ijms-2345053-supplementary.pdf]

Supplementary Information

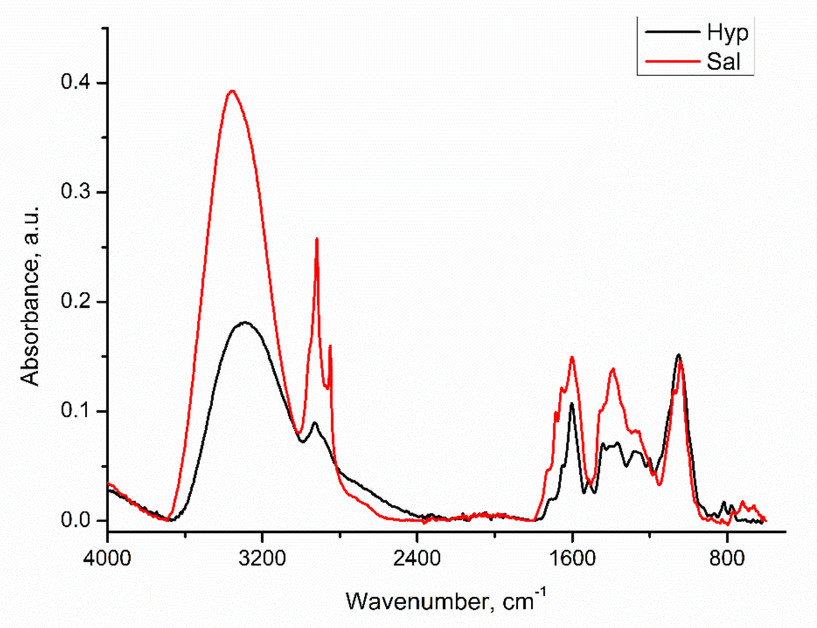

Figure S1. IR spectra of pure *H. perforatum* (Hyp) and *S. Officinalis* (Sal) extracts
